# The Effect of Polyethylene Glycol Addition on Improving the Bioconversion of Cellulose

**DOI:** 10.3390/molecules29235785

**Published:** 2024-12-07

**Authors:** Kinga Szentner, Agnieszka Waśkiewicz, Robert Imbiorowicz, Sławomir Borysiak

**Affiliations:** 1 Department of Chemistry, Faculty of Forestry and Wood Technology, Poznan University of Life Sciences, Wojska Polskiego 75, 60625 Poznan, Poland; agnieszka.waskiewicz@up.poznan.pl (A.W.); robert.imbiorowicz@icloud.com (R.I.); 2 Institute of Chemical Technology and Engineering, Poznan University of Technology, Berdychowo 4, 60965 Poznan, Poland; slawomir.borysiak@put.poznan.pl

**Keywords:** cellulose, polyethylene glycol, enzymatic reaction, FTIR, HPLC, XRD, crystallinity

## Abstract

In recent years, many studies have focused on improving the bioconversion of cellulose by adding non-ionic surfactants. In our study, the effect of the addition of a polymer, polyethylene glycol (PEG 4000), on the bioconversion of different cellulose materials was evaluated, focusing on the hydrolysis efficiency and structural changes in pure cellulose after the enzymatic hydrolysis process. The obtained results showed that the addition of non-ionic surfactant significantly improved the digestibility of cellulosic materials. The highest hydrolysis efficiency was observed for Sigmacel 101 (Cel-S101) cellulose, which consists mainly of amorphous regions. In the case of Avicel cellulose (Cel-A), PEG had a lesser effect on the bioconversion’s efficiency due to limited access to the crystal structure and limited substrate–cellulase interactions. The consistency of the obtained results is confirmed by qualitative and quantitative analyses (XRD, FTIR, and HPLC). Our findings may be helpful in further understanding the mechanism of the action of surfactants and improving the enzymatic hydrolysis process.

## 1. Introduction

**Figure 1:**
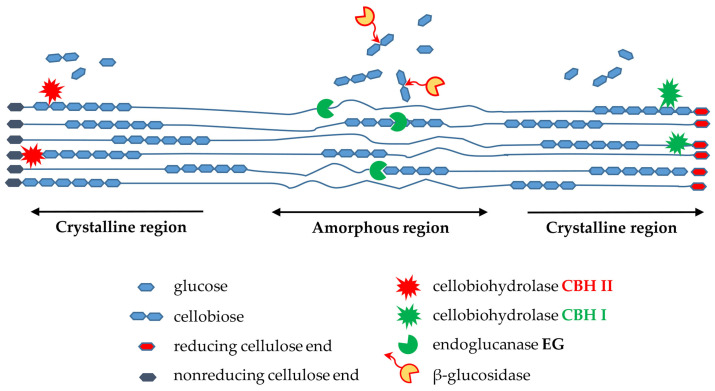
Schematic diagram of action of cellulase on cellulose material.

Cellulose is one of the most abundant polysaccharides, consisting of β,D-anhydroglucopyranose units linked by β-1,4-glycosidic bonds. The structure of cellulose is characterized by the presence of intra- and intermolecular hydrogen bonds, which allow van der Waals forces to interact between molecules [1]. In addition, cellulose microfibrils contain both ordered (crystalline) and disordered (amorphous) areas. Crystalline areas in cellulose are stable due to the numerous hydrogen bonds that occur between adjacent hydroxyl groups. In amorphous areas, the number of inter-chain hydrogen bonds is much smaller. The proportions of crystalline and amorphous areas in cellulosic materials vary depending on the source and extraction method of a given material. This has a significant impact on the chemical properties of cellulose and makes it a very attractive raw material with a variety of applications in research and industrial practice. These studies focus on, cellulose hydrolysis, in particular using enzymes (bioconversion). The process is used, for example, for the production of paper, bioplastics, textiles, nanocellulose or biofuels, as well as in medicine and polymer chemistry [2,3,4,5].To ensure the efficient enzymatic hydrolysis of cellulose the synergistic action of three major classes of enzymes is required, i.e., endo-1,4-β-D-glucanases [EG], exo-1,4-β- cellobiohydrolases (CBH), and β-D- glycosidases, which need to act in a strictly coordinated manner [6,7,8,9,10]. Endo-β-1,4-glucanases provide random cleavage of β-1,4 glycosidic bonds in the internal (amorphous) areas of cellulose, in this way are created new oligosaccharide chains of varying lengths [11]. In turn, the action of exo-1,4-β-D-glucanases (CBH) involves the removal of subunits from both reducing and non-reducing ends of the cellulose chain. As a consequence, progressively shorter polymer chains are formed, while cellobiose and glucose units are released. These enzymes digest both amorphous and crystalline cellulose, but it is the only one for the efficient degradation of crystalline cellulose (Figure 1). The accumulation of cellobiose inhibits endoglucanase enzymes, while the presence of β-glucosidases is important for complete hydrolysis of cellulose.

The effective action of enzymes also depends on the conditions of the hydrolysis reaction (temperature, reaction environment, substrate concentration, and reaction time), as well as the availability of the bioconverted material. The course of hydrolysis may vary depending on the structure of the material, its porosity, crystallinity or the degree of polymerization [12,13,14,15,16,17,18].

In recent years, many studies have focused on improving the bioconversion of cellulose. This concerns both the modification of cellulose using ionic liquids, the process of its mercerization, as well as the use of various activators, including metals, proteins, or surfactants [19,20,21,22]. Among the above-mentioned activators, surfactants are interesting. The most commonly used include Tween as a non-ionic surfactant, e.g., Tween 20, Tween 60 and Tween 80, as well as polyethylene glycol (PEG) of various molecular weights, such as PEG 2000, PEG 4000, PEG 6000, PEG 8000, and PEG 10000 [23,24,25,26,27,28,29,30,31,32]. Previous studies on the use of these compounds in enzymatic hydrolysis reactions have mainly concerned pre-processed lignocellulosic materials [22,31]. However, their efficiency varies, depending on the conditions of biomass processing and the conditions of the reaction. It was found that the addition of surfactant, especially PEG 4000, to the reaction medium improves the efficiency of cellulases. It can prevent unproductive adsorption of cellulases on the lignin fraction. Moreover, these compounds can change the composition or structure of the substrate by removing part of the lignin and increasing the availability of cellulose [21]. Initial studies showed that PEG has an effect only on the substrate with lignin. This was contradicted by other studies, which showed the effectiveness of PEG surfactant on the bioconversion of both cellulosic material after delignification and for pure cellulose. Reports on the bioconversion of pure cellulose are few and do not fully explain this process [23,24,27]. Therefore, many authors discuss the effectiveness of PEG in cellulose bioconversion using the example of cellulosic material after biomass treatment, which still contains trace amounts of lignin or hemicelluloses.

Previous studies indicate how important the addition of PEG is to the hydrolysis reaction medium, which significantly affects the catalytic efficiency of the enzyme, accelerating bioconversion by reducing cellulase inactivation [22]. It was also found that the effectiveness of the surfactant depends on its ability to bind to various substrates. Therefore, it is important that in addition to surfactants, appropriate conditions of the bioconversion process, the structural conditions of the material should be taken into account in the assessment of the process [33,34,35].

Previous studies, mainly comparative studies of cellulosic materials from biomass and pure cellulose, indicate PEG 4000 as the most effective compound that has a positive effect on enzyme activity.

In addition, it was found that surfactants can form reversible micelles that can protect the enzyme from denaturation [34].

It has been shown that the addition of a surfactant can increase the activity of cellobiohydrolase (CBH) and endoglucanase (EG). In addition, the addition of PEG can contribute to the effect on the desorption of enzymes from various substrates.

Surfactants such as PEG, e.g., PEG 4600, can also increase cellulose hydrolysis by reducing the deactivation of cellulose induced by shear force and the air–liquid interface [36]. In addition, they can also effectively help eliminate amorphous cellulose and can improve cellulase activity.

In the studies to date, despite important information on the action of PEG 4000, there is no comparison of qualitative and quantitative analysis, taking into account pure cellulose materials differing in the share of crystalline and amorphous areas. This is of importance in the context of explaining the effectiveness of enzymes with PEG.

Research on the use of PEG as a surfactant in the technological process [36] indicates the advisability of conducting further analyses to assess its usefulness. It is important to extend the current research on pure, model cellulose substrates and to assess the effect of the addition of the polymer, polyethylene glycol (PEG 4000), on the efficiency of their bioconversion and structural changes in the cellulose material after the enzymatic hydrolysis process with and without PEG.

## 2. Results and Discussion

### 2.1. Analysis of Cellulose Hydrolysis Products by High-Performance Liquid Chromatography

**Figure 2:**
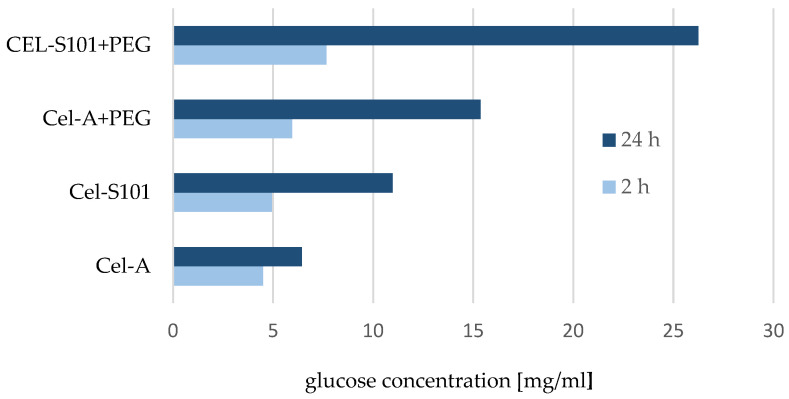
The effect of polyethylene glycol addition on the efficiency of enzymatic hydrolysis of unmodified cellulosic substrates using an enzyme complex; cellulose samples without added PEG: Cel-A, Cel-S101; cellulose samples with PEG addition: Cel-A+PEG, Cel-S101+PEG.

One of the techniques used to determine the efficiency of the hydrolysis reaction using enzymes and polyethylene glycol is liquid chromatography, which allows qualitative and quantitative determination of the reaction products formed. In the case of cellulose hydrolysis, the expected reaction product is glucose. Figure 2 shows changes in glucose contents depending on the duration of the bioconversion process, as well as the type of used material, focusing on the PEG addition to the reaction medium.

The obtained results show the glucose concentration during the reaction—at the beginning of the hydrolysis process (after 2 h) and at the end (after 24 h). The glucose content after 2 h of reaction for tested cellulose materials without added PEG was approximately 5 mg/mL. After 24 h bioconversion with Cel-S101, a further increase in glucose concentration was observed from 4.61 to 10.73 mg/mL, corresponding to a yield of 21.46%. In turn, the smallest increase in glucose level during the extended duration of the reaction was recorded in the case of Cel-A cellulose from 4.5 to 6.63 mg/mL (yield 12.06%). A slight increase in glucose concentration for Cel-A cellulose results from the presence of many crystalline areas, which makes it a slower bioconverting material.

The obtained results are consistent with the literature data that explain the reduced efficiency of the process by the inhibiting effect of the formed cellobiose [37]. The aim of our study was to increase the efficiency of the hydrolysis reaction by adding PEG. The obtained results indicate a significant increase in glucose concentration for both tested materials compared to pure cellulose.

Based on the glucose concentration, the level of which increased by approx. 50–60% after only 2 h of reaction for cellulose samples with PEG addition, a significant effect of this surfactant on the efficiency of the hydrolysis process can be seen.

In addition, after 24 h of the reaction with PEG addition, an increase in glucose concentration was observed for both cellulose substrates used. The highest glucose content was noted for Cel-S101 cellulose (26.26 mg/mL, yield 52.52%) and the lowest for Cel-A cellulose (15.37 mg/mL, yield 30.74%), which indicates a more than twofold increase in the efficiency of the hydrolysis reaction after the addition of surfactant. A similar effect of a significant increase in the efficiency of the hydrolysis reaction was noted in the studies of Ouyang et al. (2010), where after adding PEG4000, the conversion of Avicel PH101 using Celluclast and Novozyme 188 increased by 91% (from 41.1 to 78.9%) and in the case of Celluclast cellulase by 27.5% [24]. Similarly to our studies, the dependence of conversion on the type of cellulose material used was observed. The obtained results confirm earlier observations that amorphous areas in cellulose are more susceptible to enzyme action than crystalline ones [38,39]. 

Changes in glucose concentration observed in the chromatographic analysis indicate that Cel-S101 cellulose is more amorphous and for this reason the first stage hydrolysis of this material is more dynamic. This provides a more satisfactory effect of the reaction when PEG is used. This improvement and a greater amount of obtained product may be explained by the properties of polyethylene glycol. This polymer, due to numerous hydroxyl groups, is easily soluble in water and in this form can be used in the enzymatic hydrolysis reaction [32]. After dissolution, PEG is attached to cellulose through covalent bonds and in this way a bond with cellulose is formed, facilitating the adsorption of protein on the surface of the insoluble reagent [40].

Our results show the importance of an appropriate selection of reaction conditions and supramolecular structure of cellulose for the yield of the hydrolysis reaction. Similar results were obtained by Hsieh et al. [23] showing that the addition of PEG increased the efficiency of the hydrolysis reaction by 45% and the process was dependent on the cellulose substrate used. In addition, the authors provide various possible explanations for this phenomenon, including the ability of PEG to increase cellulases stability, reduce unproductive cellulase adsorption to the substrate, and increase enzyme desorption from the substrate [23]. In turn, other studies have shown a strong positive correlation between the improvement of hydrolysis with PEG addition and enzyme desorption from substrates, which depended on the ability of the surfactant to bind to different substrates. It follows that the substrate composition is a key factor influencing PEG adsorption efficiency [32]. Moreover, the addition of a surfactant to the enzymatic reaction medium influences the catalytic efficiency of the enzyme, accelerating the hydrolysis of cellulose by reducing the inactivation of cellulase [22]. 

Another equally important aspect is the understanding of the changes occurring in the cellulose fiber modified with polyethylene glycol. There are few scientific reports on the extensive topic of the mechanism of action of this surfactant on cellulose fibers. Therefore, in order to further verify the effect of polyethylene glycol on the polymorphic structure of various types of cellulose during enzymatic hydrolysis, material samples were subjected to qualitative analyses.

### 2.2. Analysis of Structural Changes in Cellulose by Infrared Spectroscopy

**Figure 3:**
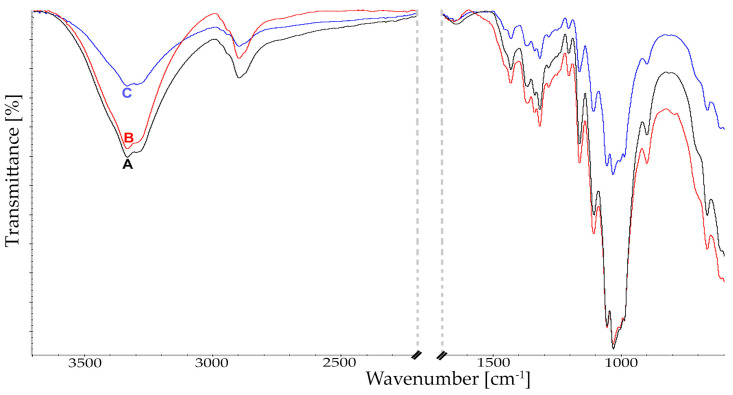
FTIR spectra of Cel-A cellulose. A−control; B−after enzymatic treatment; C−after enzymatic treatment with PEG addition.

**Figure 4:**
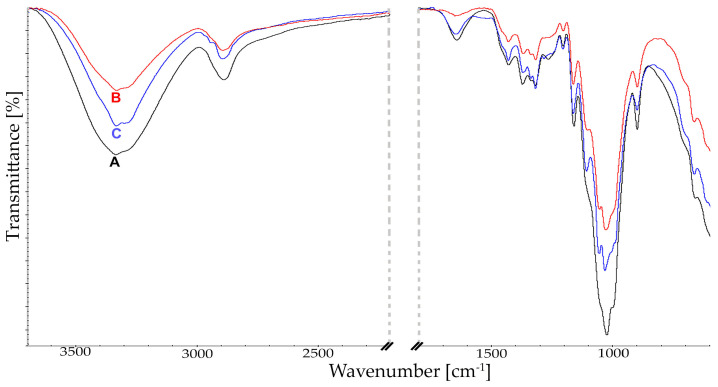
FTIR spectra of Cel-S101 cellulose. A−control; B−after enzymatic treatment; C−after enzymatic treatment with PEG addition.

A detailed analysis of qualitative changes by Fourier transform infrared spectroscopy (FTIR) was conducted for samples with the highest level of bioconversion, selected in the HPLC analysis (Figure 2). Results of these analyses are presented in Figure 3 and Figure 4. For all analyzed cellulosic materials, changes were observed in intermolecular interactions within the hydrogen bond in the range of 3500–2900 cm^−1^. Following the action of the enzyme complex with an addition of a surfactant (PEG 4000) on spectra of cellulose, a reduction was observed in band intensity at 3334 and 3286 cm^−1^, responsible for stretching vibrations of hydroxyl groups OH, as well as methyl and methylene vibrations at 2892 cm^−1^ (Figure 3 and Figure 4). Additionally, at 3334 cm^−1^ a narrowing was observed for the band width, as well as a shift towards lower values of the wave number. This indicates changes within both intermolecular and intramolecular interactions.

Analysis of infrared spectroscopic spectra also showed significant changes within the fingerprint region. It was found that depending on the cellulose material used, a slightly different course of intensity of the individual bands was observed. These changes are presented in Figure 3 and Figure 4. In the spectra for Cel-A, a reduction is observed in the intensity of most bands compared to the control sample of cellulose (Figure 3).

The recorded changes following the addition of PEG are found mainly in the crystalline and amorphous regions of cellulose and the glycoside bond. The band intensity was decreased at 1428 cm^−1^ corresponding to symmetric bending vibrations of CH2 at C-6, as well as OCH in the plane, determining also changes in the energy of hydroxyls at positions 3 and 6 (C3-3 and C6-O6) [41].

Changes in intensity were also observed in bands at 895 cm^−1^, corresponding to stretching vibrations of COC, as well as CCO and CCH at C-5 and C-6, and bending vibrations of C1-H. Moreover, after the addition of polyethylene glycol to the reaction medium the intensity of bands within the glycoside bond at 1160 cm^−1^ was reduced (Figure 3). Following the reaction of hydrolysis without PEG, these changes were not as marked. This indicates less efficient bioconversion of microcrystalline cellulose as a result of the reaction run only with the addition of the enzyme complex. In the case of Cel-S101 cellulose, significant qualitative differences were found (Figure 4).

Changes for this polymer result primarily from the fact that this type of cellulose is characterized by a low degree of crystallinity due to a high share of amorphous regions [42]. This in turn enhances susceptibility to the action of enzymes and efficiency of hydrolysis. In spectra for this batch of samples, a decrease in intensity is observed compared to the control in the bending bands of CH at 1370 cm^−1^, the bands of CH2 and C-6 at 1316 cm^−1^, as well as bending vibrations of COH in the plane at C-6 for 1199 cm^−1^. This reduction in relative band intensity was also found for bending vibrations of CH2 groups at C-6 and bending vibrations of OCH at 1429 cm^−1^. Additionally, changes were also recorded within the band at 895 cm^−1^. Moreover, differences observed in the relative intensity of bands for crystalline and amorphous regions indicate changes in the crystallinity of cellulose. This is explained by the fact that the amorphous regions, being more susceptible to enzyme action, are the first to be degraded, which is followed by the degradation of ordered (crystalline) regions. The reorganization of cellulose structure is also manifested in quantitative analyses (Figure 2), which show that amorphous regions are more effectively digested by cellulolytic enzymes. The effect of the enzyme complex containing PEG on structural changes in cellulose also reflects vibrations of other groups for this polymer. Thus, differences were recorded in band intensity of asymmetric stretching vibrations of the C-O-C glycoside bond, as well as bending vibrations of OH in C-OH at 1165 cm^−1^, indicating both depolymerization of the cellulose chain and the interaction of PEG with cellulose. Moreover, it may be assumed that—as it was reported by Li [21]—Cel-S101 cellulose, thanks to its porous and disordered structure, facilitates the bonding of the enzyme with the polymer. The addition of PEG also influenced the catalytic efficiency of the enzyme, accelerating hydrolysis of cellulose [22]. Following the bioconversion reaction differences in band intensity were also observed for stretching vibrations of C-O at C-3 and C-C at 1058 cm^−1^. Presented spectra indicate a significant difference in intensity and a shift towards lower values of these bands (Figure 4).

The FTIR spectra obtained for the non-modified material confirmed that enzymatic treatment of cellulose, both with and without the addition of PEG, results in its structural changes. This analysis indicates a different ordering of the material following hydrolysis both within the crystalline and amorphous regions, which in turn is manifested in crystallinity of cellulose. The obtained results confirm that amorphous regions are more effectively digested by cellulases, as shown by the apparent increase in crystallinity of cellulose after the hydrolysis reaction. Moreover, FTIR qualitative analysis confirms the mechanism of enzymatic action. Crystalline cellulose of limited accessibility is hydrolyzed mainly by exoglucanases. In turn, endoglucanases requiring greater polymer accessibility hydrolyze primarily amorphous regions of the polymer, leading to a reduction in molecular mass. Observed qualitative changes may provide valuable information indicating further use of a given cellulosic material.

### 2.3. XRD Investigation of Cellulose Following the Use of Polyethylene Glycol

X-ray diffraction (XRD) analyses were conducted to assess changes in the supramolecular structure of cellulosic materials following enzymatic hydrolysis with and without PEG.

**Figure 5:**
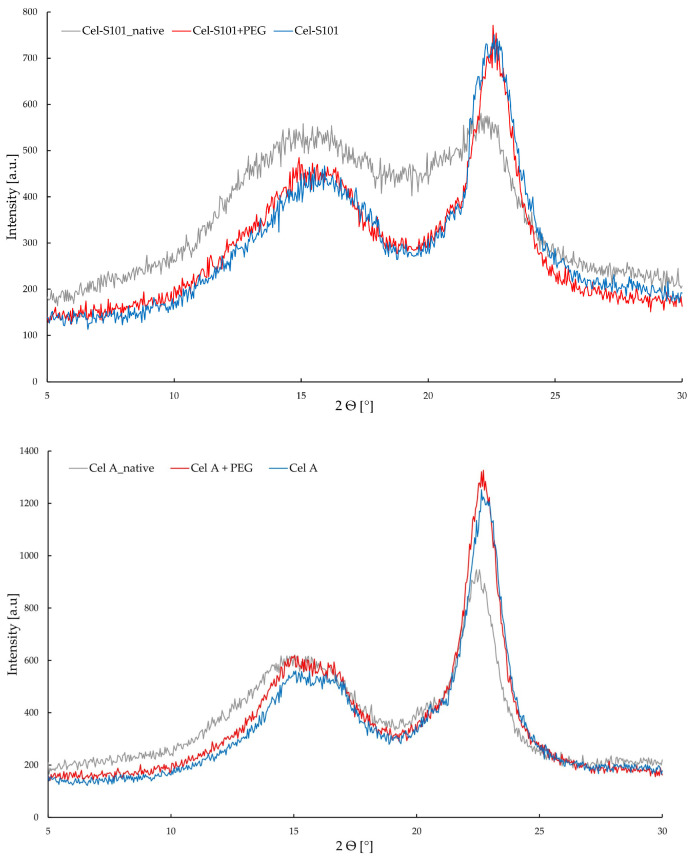
XRD patterns of cellulose: unmodified (Cel-A-native and Cel-S101-native), after reaction with enzyme (Cel-A and Cel-S101) after enzyme + PEG modification (Cel-A+PEG and Cel-S101+PEG).

Figure 5 shows diffraction patterns of cellulosic materials before and after bioconversion with and without the addition of PEG. The diffractograms of all cellulose materials showed peaks characteristic peaks at 2Θ = 15°, 17° and 22.7°, coming from polymorphic forms of cellulose I [43]. These peaks come from planes with Miller indices (1–10), (110), and (200), respectively [44]. It should be emphasized that the obtained X-ray patterns are characterized by significant variation in intensity values, which indicates changes in the degree of crystallinity. It was shown that the enzymatic treatment process with and without PEG resulted in a significant increase in the crystallinity content of cellulose (Table 1). 

This is particularly noticeable for cellulose Cel-S101 for which an increase in crystallinity of c.a. 60% was observed. The changes in the degree of crystallinity for both tested systems indicate that the cellulose bioconversion process takes place mainly in amorphous parts. Enzymatic hydrolysis of amorphous cellulose causes the crystalline cellulose fraction to remain in the tested samples, which is much more resistant to the action of enzymes. It may indicate the high activity of endoglucanases, which at the initial stage of hydrolysis are responsible for the decomposition of amorphous regions of cellulose. Similar results were obtained during acid hydrolysis [45], as well as hydrolysis using ionic liquid [46,47]. Moreover, our results confirm findings of [48] indicating differences in the energy balance between amorphous and crystalline regions in cellulose. It is worth emphasizing that the efficiency of the reaction after PEG addition during the enzymatic reaction of cellulose depends on the structural conditions of the substrate. Its addition affects the effective action of cellulases in both crystalline and amorphous regions. An interesting observation is the fact that the presence of PEG during the enzymatic reaction of cellulose does not affect the degree of crystallinity. However, HPLC studies have shown a significant effect of PEG on the efficiency of glucose formation. This may mean that although PEG does not affect the interaction in the supramolecular structure of cellulose, it can affect the hydrolysis process of shorter-chain sugars (oligomers), which can be formed as a result of the effective action of cellobiohydrolases (CBH). Polyethylene glycol can therefore positively affect the activity of cellulases and increase the bioconversion of cellulose. The obtained results constitute an important complement to both HPLC chromatographic analysis and FTIR spectroscopy. 

## 3. Materials and Methods

### 3.1. Materials

Two different cellulose materials were used in the study. Namely micrometric cellulose Avicel with an average particle size of 50 µm (Cel-A) and micrometric cellulose Sigmacell Type 101 (Cel-S101) with an average particle size of 18 µm. All cellulose materials were purchased from Sigma-Aldrich (Poznan, Poland).

This study used cellulases from *Trichoderma reesei* ATCC 26.921 with activity of 700 U/g purchased from Sigma-Aldrich (Poznan, Poland). All reagents used for reaction, e.g., buffers, polyethylene glycol, and sodium hydroxide, were purchased from Sigma-Aldrich (Poznan, Poland). The cellulase complex for the hydrolysis reaction was prepared by mixing the enzyme extract with a citrate buffer solution (50 mM) in a volume ratio (enzyme/buffer) of 1:50.

### 3.2. The Enzymatic Treatment of Cellulose

The hydrolysis reaction process was carried out in two variants, with the use of a surfactant PEG 4000 and without it. First, 500 mg of microcrystalline celluloses (Avicel, Sigmacel 101) was supplemented with 10 mL of 50 mM citrate buffer solution with pH = 4.8. First, pre-incubation of the samples was run for 60 min at 50 °C with sample shaking at 150 rpm/min (Incubated Shaker (IST-3075, Lab Companion, Seoul, JeioTech, Republic of Korea).

Then, 5 mL of the enzyme/citrate buffer complex (1:50) was added to the test tubes of the first reaction variant. Additionally, in the second variant an appropriate amount of PEG (0.05 g/g glucan) was added.

The reaction continued for 2 h. Next 5 mL of the cellulase complex solution was added. The reaction mixture was shaken at 240 rpm at 50 °C and pH 4.9 at two time points (2 h and 24 h). To complete the hydrolysis reaction, samples were heated for 5 min at 100 °C, then frozen and stored at −20 °C until further analysis. After thawing, the clear liquid was filtered through syringe filters (Chromafil PET 0,45 µm, Macherey-Nagel GmbH & Co. KG, Dueren, Germany) into chromatography vials. The remaining solid was washed 10 times with deionized water to remove residual protein. Then, the cellulose material was dried in a laboratory dryer at 60 °C. The samples were stored in a desiccator over anhydrous P_2_O_5_ and left for further analysis (X-ray and IR spectra).

### 3.3. Analysis of Cellulose Hydrolysis Products by Liquid Chromatography (HPLC/RI)

Qualitative and quantitative analysis of the main product of cellulose bioconversion—glucose—was performed using high-performance liquid chromatography combined with refractometric detection (HPLC/RI). Before performing chromatographic analysis, the samples were centrifuged at 0 °C for about 20 min and then the obtained supernatant was filtered through syringe filters (Chromafil PET 0,45 µm, Macherey-Nagel GmbH & Co. KG, Dueren, Germany). Chromatographic analysis was performed using a liquid chromatograph (2695 Waters, Milford, MA, USA) connected to a refractometric detector (2414, Waters, USA) and a chromatographic column (Aminex HPX-87, Bio-Rad, Hercules, CA, USA). The separation parameters were as follows: mobile phase sulfuric acid (VI) at a concentration of 0.004 mol/L, flow rate 0.6 mL/min, oven temperature 65 °C, injection volume 5–10 µL. The results were processed using Empower TM 1 software, using an appropriate calibration curve. Each analysis was performed in triplicate.

### 3.4. X-Ray Diffraction Analysis (XRD)

The XRD technique was used to determine the changes in the degree of crystallinity of the enzymatically hydrolyzed and polyethylene glycol-modified cellulose material. The measurements were performed using a SmartLab X-ray diffractometer (Rigaku, Tokyo, Japan) applying Kα radiation from a lamp with a Cu-Kα anode (1.5418 Å).

The anodic voltage and current were equal to 40 kV and 30 mA, respectively. The recording was carried out in the 2θ angular range of 5–30°, in the step of 0.04°. The deconvolution of the obtained peaks was performed using the method developed by Hindeleh and Johnson [49]. After separation of the X-ray diffraction lines, the degree of crystallinity (Xc) was determined by comparing the areas under the crystalline peaks and the amorphous curve, using the formula described in the report [50,51].

### 3.5. Analysis of Structural Changes in Cellulose by Infrared Spectroscopy (ATR-FTIR)

ATR-FTIR spectroscopy analysis was used as a non-destructive method to assess structural changes in the bioconverted cellulosic material. Approximately 1 mg of sample was used for analysis, previously crushed, homogenized, and dried in a vacuum over P_2_O_5_. The spectra were recorded using a Nicolet iS5 FTIR spectrometer (Thermo Fisher Scientific, Waltham, MA, USA) with the iD7-ATR attachment, in the range from 4000 to 450 cm^−1^ at a resolution of 4 cm^−1^, recording 32 scans.

The analysis of the obtained basic spectra was performed using the Omnic 9 software (Thermo Scientific). Each sample was tested three times.

## 4. Conclusions

This study compared the conversion of various cellulosic materials in the presence of PEG. Summing up the obtained results, it was stated that an addition of the non-ionic surfactant considerably enhanced digestibility of cellulosic materials.

It was shown that enzymatic hydrolysis using cellulases and PEG was much more efficient during 24 h incubation in the presence of polyethylene glycol. This confirms not only advisability of PEG addition, but also the significance of duration of the hydrolysis process. The highest hydrolytic efficiency was recorded for Cel-S101 cellulose. This polymer is mostly composed of amorphous regions. For this reason, the hydrophobic part of synthetic polyethylene glycol may fill the spaces, forming a coating protecting the enzyme against adverse protein denaturation [21]. The situation was different in the case of Avicel microcrystalline cellulose. Due to its compact structure and absence of void spaces, to which PEG might attach, this material is less accessible to the action of cellulases. Nevertheless, the addition of PEG improved the activity of cellulases and consequently increased the bioconversion of both cellulose materials.

Both qualitative and quantitative analyses (XRD, FTIR, and HPLC) show consistent results and supplement conclusions concerning the enzymatic bioconversion process. Recorded diffraction patterns and spectra also showed that efficiency of bioconversion is dependent on the type of the substrate, on which enzymatic hydrolysis is run. The course of the reaction is determined by the share of crystalline and amorphous regions in the material, as well as particle size and surface accessibility. These factors explain the most efficient action of cellulases with the addition of PEG for Cel-S101 cellulose. Our findings may be helpful in further understanding the mechanism of action of surfactants and improving the enzymatic hydrolysis process.

## Figures and Tables

**Table 1:** Analysis of the supramolecular structure of cellulose preparations by wide-angle X-ray diffraction before and after enzymatic reaction of cellulose with and without addition PEG.

Cellulose	Crystallinity of Cellulose [%]			Increase in Crystallinity After Treatment [%]
	Before Enzymatic Reaction	After Enzymatic Reaction Without PEG	After Enzymatic Reaction with PEG	
**Cel-A**	49	65	64	c.a. 30
**Cel-S101**	32	51	52	c.a. 60

## Data Availability

Data are available from the corresponding author upon reasonable request.
